# DBC1/CCAR2 is involved in the stabilization of androgen receptor and the progression of osteosarcoma

**DOI:** 10.1038/srep13144

**Published:** 2015-08-07

**Authors:** Sajeev Wagle, See-Hyoung Park, Kyoung Min Kim, Young Jae Moon, Jun Sang Bae, Keun Sang Kwon, Ho Sung Park, Ho Lee, Woo Sung Moon, Jung Ryul Kim, Kyu Yun Jang

**Affiliations:** 1 Department of Orthopedic Surgery, Chonbuk National University Medical School, Research Institute of Clinical Medicine of Chonbuk National University-Biomedical Research Institute of Chonbuk National University Hospital and Research Institute for Endocrine Sciences, Jeonju, Republic of Korea; 2 Program in Nano Science and Technology, Department of Transdisciplinary Studies, Seoul National University Graduate School of Convergence Science and Technology, Suwon, Korea; 3 Department of Pathology, Chonbuk National University Medical School, Research Institute of Clinical Medicine of Chonbuk National University-Biomedical Research Institute of Chonbuk National University Hospital and Research Institute for Endocrine Sciences, Jeonju, Republic of Korea; 4 Department of Preventive Medicine, Chonbuk National University Medical School, Jeonju, Republic of Korea; 5 Department of Forensic Medicine, Chonbuk National University Medical School, Jeonju, Republic of Korea

## Abstract

Deleted in breast cancer 1 (DBC1/CCAR2) is a protein of interest because of its diverse roles in tumorigenesis and its possible role as an androgen receptor (AR) co-activator. However, there are limited studies on the role of DBC1 in osteosarcoma. Therefore, we investigated the role of DBC1 and AR and their relationship in osteosarcoma. Immunohistochemical expression of DBC1 and AR was significantly associated with higher clinical stage and higher histologic grade, and predicted shorter survival. Especially, DBC1 expression was an independent prognostic indicator of overall survival (*p* = 0.005) and relapse-free survival (*p* = 0.004) by multivariate analysis. In osteosarcoma cell lines, U2OS and SaOS2, the knock down of DBC1 and AR with siRNA significantly reduced cellular proliferation and inhibited proliferation-related signaling. In addition, the knock down of DBC1 and AR decreased the invasion activity and inhibited invasion-related signaling of osteosarcoma cells. Interestingly, DBC1 affects the stabilization of AR protein *via* a mechanism involving the ubiquitination of AR. Proteosome-mediated degradation and poly-ubiquitination of AR were increased with the knock-down of DBC1. In conclusion, this study has shown that DBC1 is involved in the stabilization of AR protein and DBC1-AR pathways might be involved in the progression of osteosarcoma.

Osteosarcoma is the most common primary malignant bone tumor^1^. It usually occurs during childhood and adolescence in the metaphysis of long bones, including large growth plates, and it demonstrates high proliferation activity and bone turnover^2^. The mechanisms involved in the development of osteosarcoma are not clear and various factors such as sex, age, genetic, and hereditary factors have been associated with the development of osteosarcoma^3^,^4^.

Deleted in breast cancer 1 (*DBC1/CCAR2)* gene was named based on its deletion in breast cancer^5^. The role of DBC1 has primarily been thought to be its inhibitory role of SIRT1 and it has been suggested as a tumor suppressor^6^,^7^. However, increased expression of DBC1 was associated with poor prognosis of the cancer patients^8^,^9^,^10^,^11^,^12^,^13^,^14^,^15^,^16^. Moreover, depletion of DBC1 induced apoptosis of tumor cells and inhibited the proliferation of cancer cells^15^,^17^,^18^,^19^,^20^. In addition, it has been suggested that DBC1 might be involved in the progression of hormone receptor-related human malignant tumors^12^,^14^,^15^. DBC1 promoted the survival of breast cancer cells by modulating estrogen receptor α and β^21^,^22^. Recently, a cooperative role of DBC1 with androgen receptor (AR)^19^ and concomitant expression of DBC1 and AR in advanced human malignant tumors have been reported^9^,^10^,^11^.

AR is a member of the nuclear receptor family and acts as a ligand regulated transcription factor^23^. The expression of AR had been reported in various types of normal and malignant tissues^24^,^25^, such as breast cancer^26^, gastric cancer^27^, hepatocellular carcinoma^24^, clear cell renal cell carcinoma^10^, lymphoma^28^,^29^, and soft-tissue sarcomas^11^. The activation of AR could be mediated by various co-activators, and recently it has been demonstrated that DBC1 serves as a co-activator of AR^19^.

Recently, there is an increasing number of reports showing that DBC1 and/or AR are involved in the progression of various human malignant tumors, including soft-tissue sarcomas^11^. However, there are limited reports investigating the role of DBC1 in human osteosarcoma. Therefore, this study investigated the role of DBC1 and AR in osteosarcoma by using human osteosarcoma tissue samples and osteosarcoma cell lines.

## Results

### The expression of DBC1 and AR in human osteosarcoma patients

**Figure 1:**
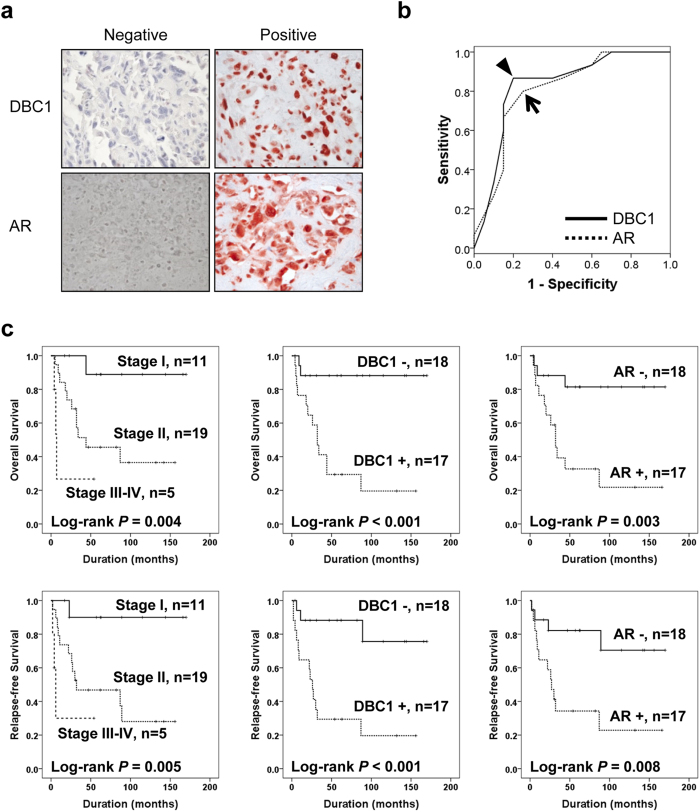
Immunohistochemical expression of DBC1 and AR in human osteosarcoma tissue and Kaplan-Meier survival analysis in 35 osteosarcoma patients. (**a**) DBC1 is expressed in the nuclei of osteosarcoma cells and AR is expressed both in the nuclei and cytoplasm of osteosarcoma cells. (**b**) Statistical analysis of the sensitivity and specificity of the DBC1 and AR immunohistochemical staining scores for the death of the patient (event of overall survival) by receiver operator characteristic curves. The arrow head indicate the cut-off point for the immunohistochemical staining score of DBC1 and an arrow indicates the cut-off point for AR immunostaining. (**c**) Overall survival and relapse-free survival are shown according to the tumor stage, DBC1 expression, and AR expression.

In human osteosarcoma tissues, the expression of DBC1 was mainly localized in the nuclei of tumor cells and AR was expressed both in the nuclei and cytoplasm (Fig. 1a). The cut-off points for the immunohistochemical staining scores (IHC scores) of DBC1 or AR immunostaining were determined at the most likely point for the prediction of death of osteosarcoma patients. The most likely points had the highest area under the curve (AUC) in receiver operating characteristic curve analysis. The cut-off points for the IHC scores of DBC1 and AR were 12 and 11, respectively. The immunostaining for DBC1 was considered positive when the IHC score for DBC1 was equal or greater than 12 (AUC, 0.833; *p* < 0.001; 95% confidence interval [95% CI], 0.689–0.978). The immunostaining for AR was considered positive when the IHC score was equal or greater than 11 (AUC, 0.775; *p* = 0.006; 95% CI, 0.612–0.938) (Fig. 1b). In osteosarcoma patients, 49% (17/35) and 49% (17/35) of cases were included in DBC1-positive and AR-positive groups, respectively. As shown in Table 1, DBC1-positivity was significantly associated with larger tumor size (*p* = 0.028), higher tumor stage (*p* = 0.035), and higher histologic grade (*p* = 0.015). AR-positivity was significantly associated with higher tumor stage (*p* = 0.005) and higher histologic grade (*p* = 0.002). In addition, there was a significant association between DBC1-positivity and AR-positivity (*p* = 0.001). When we evaluated the correlation between the IHC scores of DBC1 expression and AR expression, there was a significant correlation (Spearman’s rho; 0.577, *p* < 0.001).

On univariate survival analysis, tumor size, tumor stage, the presence of distant metastasis, histologic grade, and the expression of DBC1 and AR were significantly associated with both overall survival (OS) and relapse-free survival (RFS) (Table 2 and Fig. 1c). The osteosarcoma patients included in the DBC1-positive group showed an 8.639-fold (95% CI; 1.940–38.469, *p* = 0.005) greater risk of death and a 6.657-fold (95% CI; 1.875–23.629, *p* = 0.003) greater risk of relapse or death compared with the DBC1-negative group. The expression of AR also predicted a 5.551-fold (95% CI; 1.551–19.864, *p* = 0.008) greater risk of death and a 4.201-fold (95% CI; 1.336–13.216, *p* = 0.014) greater risk of relapse or death. Multivariate analysis was performed with the inclusion of the factors significantly associated with OS or RFS by univariate analysis. Multivariate analysis revealed DBC1-positivity as an independent prognostic indicator of OS and RFS. The expression of DBC1 predicted an 8.639-fold (95% CI; 1.940–38.469, *p* = 0.005) greater risk of death and a 6.555-fold (95% CI; 1.825–23.549, *p* = 0.004) greater risk of relapse or death. The age of the patients was a possible prognostic indicator of RFS (*p* = 0.057) by multivariate analysis (Table 2 Model 1). Because there was significant correlation between the expression of DBC1 and AR, we performed additional multivariate analysis excluding DBC1 expression and showed the expression of AR as an independent prognostic indicator of OS (*p* = 0.007) and RFS (*p* = 0.025) (Table 2 Model 2).

### Knock-down of DBC1 and AR inhibits the proliferation and invasion activity of osteosarcoma cells

**Figure 2:**
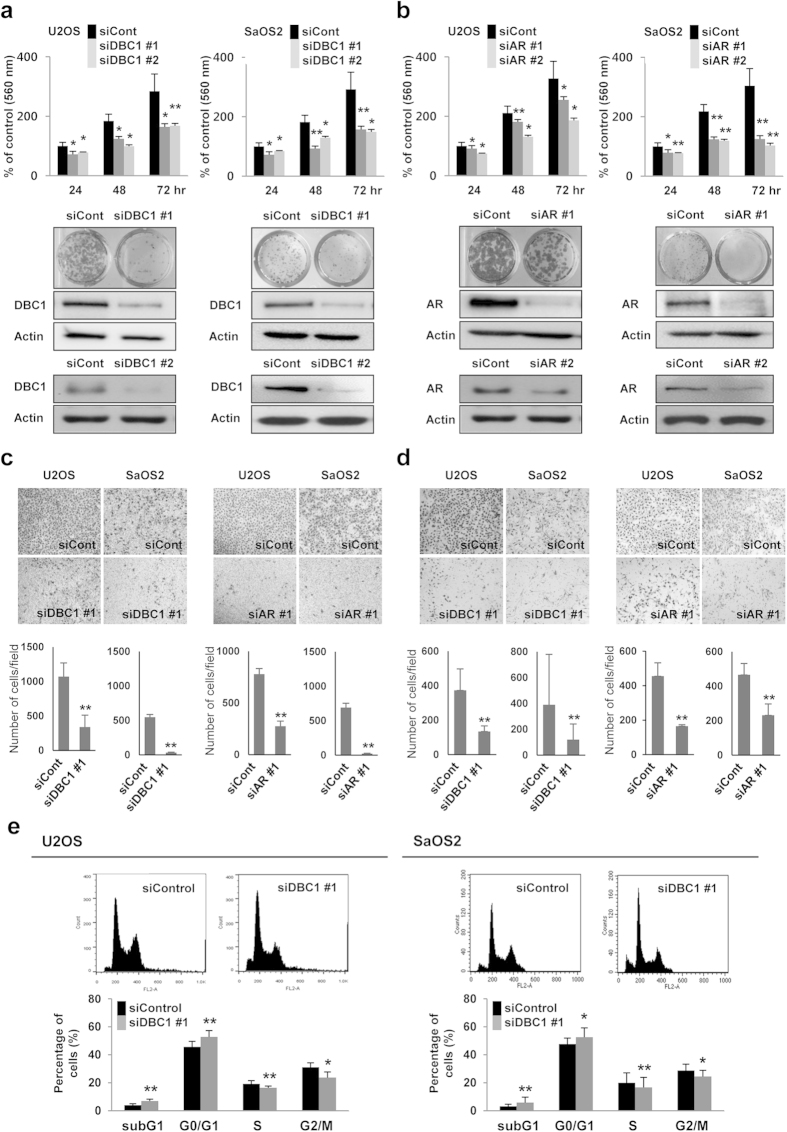
Inhibition of DBC1 and AR decrease the proliferation, migration activity, and invasion activity of osteosarcoma cells. (**a**,**b**) The knock-down of DBC1 (**a**) or AR (**b**) with two sets of siRNAs for DBC1 or AR (siDBC1 #1, siDBC1 #2, siAR #1, and siAR #2) inhibit the proliferation of both U2OS and SaOS2 osteosarcoma cells as indicated by an MTT and colony forming assay. (**c**) The knock-down of DBC1 and AR significantly reduced the migration activity of both U2OS and SaOS2 cells. (**d**) The invasion capacity of U2OS and SaOS2 cells are significantly decreased with the knock-down of DBC1 and AR in a matrigel invasion assay. (**e**) Cell cycle analysis with flow cytometry shows that the knock-down of DBC1 significantly increases the subG1 and G0/G1 populations in the both U2OS and SaOS2 cells. The statistical analysis for the cell cycle analysis was performed from the flow-cytometry analysis six times. The MTT assay was performed by seeding 1 × 10^3^ U2OS cells and 2 × 10^3^ SaOS2 cells, and the absorbance measured at 560 nm. For the colony forming assays, 1 × 10^3^ U2OS cells and 2 × 10^3^ SaOS2 cells were seeded per well of a 24-well culture plate for seven days. The trans-chamber migration and invasion assays were performed after seeding 4 × 10^4^ U2OS cells and 1 × 10^5^ SaOS2 cells. *p < 0.05, **p < 0.001.

Because clinical data suggests that the expression of DBC1 and AR may be involved in the progression of osteosarcomas despite the limited number of cases, we evaluated the effects of a knock-down of DBC1 and AR in osteosarcoma cells. The knock-down of DBC1 and AR with two sets of siRNA significantly inhibited the proliferation of both U2OS (wild-type P53) and SaOS2 (P53-null) osteosarcoma cells demonstrated by MTT and colony forming assays (Fig. 2a,b). In addition, overexpression of DBC1 increased proliferation of U2OS cells. The proliferation of DBC1-overexpressing cells decreased with the co-transfection of AR siRNA. However, the proliferation of co-transfected cells was significantly higher than in control cells (Supplementary Fig. S1). The knock-down of DBC1 and AR significantly reduced the migration and invasion activity of both U2OS and SaOS2 cells (Fig. 2c,d). The decreased proliferation of osteosarcoma cells with a knock-down of DBC1 was associated with a significant increase in the subG0/G1 population and a G0/G1 arrest in U2OS and SaOS2 (Fig. 2e). In addition, the knock-down of DBC1 increased cleavage of PARP1 and made the cells susceptible to treatment with doxorubicin, one of the conventional chemotherapeutic drugs (Supplementary Fig. S2).

**Figure 3:**
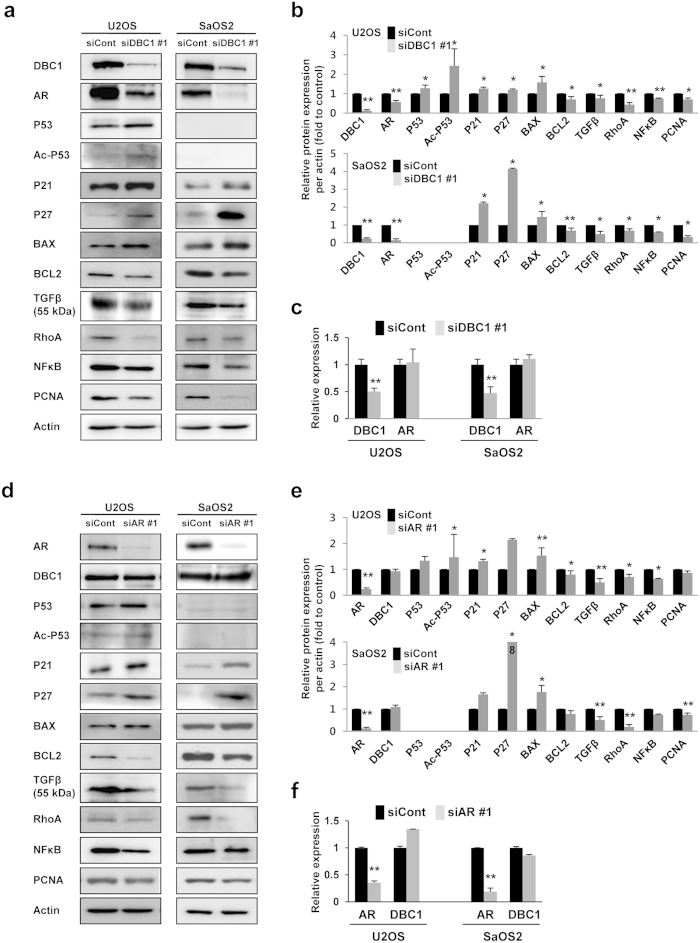
The expression of DBC1 and AR are associated with the signals for the proliferation and invasion of cells. (**a**,**b**) Western blotting indicates that the protein levels of BCL-2, TGFβ, RhoA, NFκB, and PCNA were significantly decreased and the expression of P21 and P27 were significantly increased with the knock-down of DBC1 by siRNA in the both U2OS (wild-type P52) and SaOS2 (P53-null) cells. In U2OS cells, acetylation of P53 was significantly increased with the knock-down of DBC1. Especially, the protein level of AR was significantly decreased with the knock-down of DBC1. (**c**) However, the mRNA level of AR did not decrease with the knock-down of DBC1 as demonstrated by quantitative reverse-transcription PCR. (**d**,**e**) The knock-down of AR increased the protein levels of P21, P27, and BAX, and decreased the protein levels of TGFβ, RhoA, and NFκB in the both U2OS and SaOS2 cells. But the protein level of DBC1 (**e**) and mRNA level of DBC1 (**f**) were not significantly changed with the knock-down of AR with siRNA for AR. The statistical analysis for the quantification of protein expression was performed from three western blots. *p < 0.05, **p < 0.001.

Because the expression of DBC1 and AR were associated with the proliferation and invasion activity of osteosarcoma cells, we evaluated the expression of DBC1, AR, and the molecules related with the proliferation and invasiveness of cells (Fig. 3). As previously reported, the acetylation of P53 increased with the knock-down of DBC1 in U2OS cells. In addition, the knock-down of DBC1 increased the protein expression of P21, P27, and BAX but decreased the expression of BCL2, TGFβ, RhoA, NFκB, and PCNA in both U2OS and SaOS2 cells (Fig. 3a,b). Interestingly, the protein expression of AR was decreased with the knock-down of DBC1, but the mRNA level of AR remained unchanged with the knock-down of DBC1 (Fig. 3b,c). The knock-down of AR increased the protein levels of acetylated P53, P21, P27, and BAX and decreased the expression of TGFβ and NFκB in U2OS cells. In SaOS2 cells, the protein levels of P27 and BAX were increased and the expression of TGFβ and RhoA were decreased with the knock-down of AR (Fig. 3d,e). However, the expression of DBC1 protein and DBC1 mRNA were not affected by the knock-down of AR (Fig. 3e,f).

### Transient knock-down of DBC1 by transfection of siRNA against DBC1 decreases AR expression through ubiquitination and proteosome-mediated degradation

**Figure 4:**
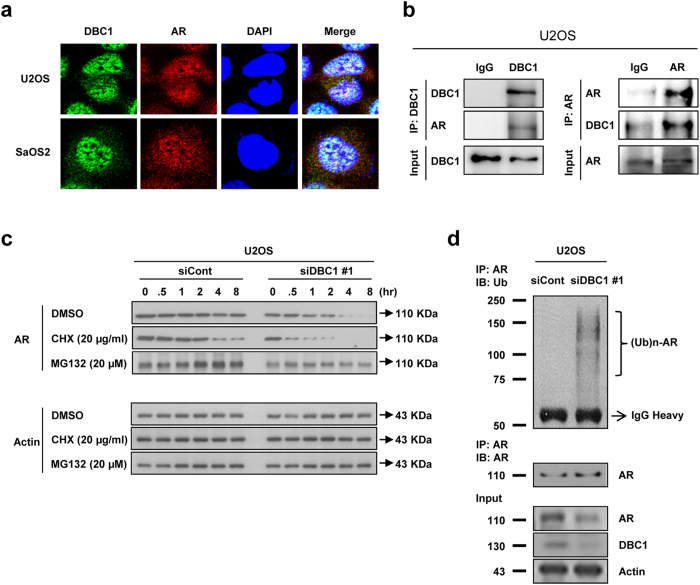
DBC1 is involved in the post-translational stabilization of AR by modulating the ubiquitination and proteosome-mediated degradation of AR. (**a**) DBC1 (green) and AR (red) are co-localized mainly in the nuclei of U2OS and SaOS2 osteosarcoma cells by the confocal microscopic image with immunofluorescence staining. (**b**) Immunoprecipitation indicates direct binding of DBC1 and AR. AR is detected in samples taken with immunoprecipitation for DBC1 and *vice versa*. (**c**) Transfection of DBC1 siRNA into U2OS cells decreases AR stability *via* a proteasome-mediated pathway. The protein level of AR was decreased more in cells transfected with DBC1 siRNA than in cells transfected with control siRNA. U2OS cells transfected with control or DBC1 siRNA for 24 hours and treated with DMSO (control vehicle), cycloheximide (CHX, 20 μg/ml), or MG-132 (20 μM) for the indicated time. (**d**) Transfection of DBC1 siRNA into U2OS cells caused ubiquitination of AR. U2OS cells were transfected with control or DBC1 siRNA for 24 hours using Lipofectamine. Subsequently, the transfected cells were treated with MG-132 (20 μM) for 4 h and total lysates of cells were immuno-precipitated with anti-androgen receptor antibodies and blotted with anti-ubiquitin antibodies.

There was significant correlation between the expression of DBC1 and AR protein levels as shown in immunohistochemical staining in human osteosarcoma tissue samples and Western blotting in osteosarcoma cells (Table 1 and Fig. 3a). However, the mRNA level of AR was unchanged with a knock-down of DBC1, despite a significant decrease in protein level of AR (Fig. 3a,b). Therefore, we investigated the interaction between DBC1 and AR proteins. Confocal analysis showed co-localization of DBC1 and AR mainly in the nuclei of osteosarcoma cells (Fig. 4a) and immunoprecipitation (IP) showed direct binding of DBC1 to AR (Fig. 4b). Because DBC1 binds directly to AR and the expression level of DBC1 affects the protein levels of AR, we hypothesized that DBC1 may be involved in the post-translational stabilization of AR and explored the mechanism of AR degradation, which occurs as a result of the transient knock-down of DBC1. AR had been shown to be degraded by ubiquitin-dependent proteosomal degradation^30^. Therefore, we first examined AR stability in U2OS cells with treatment of cycloheximide, an inhibitor of protein translation, after transfection of control siRNA or DBC1 siRNA. As shown in Fig. 4c, the stability of AR was decreased with knock-down of DBC1, as evidenced by the earlier disappearance of AR in cells transfected with DBC1 siRNA compared to the cells transfected with control siRNA. To determine whether the degradation of AR with a knock-down of DBC1 was proteosome-mediated, we utilized MG-132, a proteasome inhibitor. While AR levels decreased within 4 hours of treatment of U2OS cells with DBC1 siRNA, levels of AR did not decrease over 4 hours in U2OS cells transfected with control siRNA with MG-132 (Fig. 4c) suggesting that the decreased levels of AR caused by the transfection of DBC1 siRNA is dependent on the proteasome. To confirm that proteosome-mediated AR degradation occurs through ubiquitination, we evaluated the level of ubiquitination of AR occurring with different expression levels of DBC1. After transfection of the control or DBC1 siRNA into U2OS cells, we incubated cells with MG-132 for 4 hours and performed IP with AR antibody and immunoblotted with anti-ubiquitin antibodies. As shown in Fig. 4d, IP with AR antibody after incubation of MG-132 resulted in poly-ubiquitination of AR, while cells transfected with the control siRNA showed considerably less ubiquitination; these results are consistent with proteasome-dependent mechanism of degradation.

## Discussion

In this study, the expression of DBC1 was significantly associated with advanced clinicopathologic factors of osteosarcoma such as larger tumor size, higher tumor stage, and higher histologic grade. Moreover, DBC1 expression in osteosarcoma was an independent prognostic indicator of OS despite the limited number of cases of osteosarcoma patients. In line with these results, DBC1 expression was significantly associated with higher tumor stage, higher histologic grade, and distant metastasis in soft-tissue sarcomas^11^. Moreover, it has been reported that the expression of DBC1 is associated with poor prognosis of various human malignant tumors, including gastric carcinoma^13^, esophageal carcinomas^17^, breast carcinomas^12^,^16^, colorectal carcinomas^8^, clear cell renal cell carcinomas^10^, diffuse large B cell lymphomas^9^, ovarian carcinomas^14^, and soft-tissue sarcomas^11^. These results suggest that DBC1 is widely involved in the progression of human malignant tumors regardless of tumor types. However, the mechanism how DBC1 is involved in the progression of human cancers is poorly understood. DBC1 has Janus-faced roles in tumorigenesis: it can be tumorigenic and anti-tumorigenic. DBC1 conserves the function of tumor suppressor P53 by inhibiting SIRT1^6^,^7^. In contrast, DBC1 inhibits both SIRT1 and tumor suppressor SUV39H1 by disrupting the SUV39H1-SIRT1 complex^19^. In addition, DBC1 suppresses BRCA1^31^ and activates oncogenic E-twenty six transcription factor PEA3 (Polyoma enhancer activator 3)^16^. DBC1 also attenuated the transcriptional activity of LXRα (liver X receptor α) by forming the DBC1-LXRα complex, and RNA interference of DBC1 inhibited proliferation of MCF-7 breast cancer cells^18^. Moreover, despite the role of DBC1 for the inhibition of SIRT1, co-expression pattern of DBC1 and SIRT1 in human cancers is common, and both are associated with progressive clinicopathologic factors of human malignant tumors^10^,^11^,^12^,^13^. Similarly, despite the inhibitory role of DBC1 for BRCA1, the expression of DBC1 and BRCA1 were positively correlated and both predicted shorter survival of ovarian carcinoma patients^14^. Therefore, there is a possibility that DBC1 may be involved in tumorigenesis, independent of SIRT1.

In addition to the role of DBC1 as a negative regulator of SIRT1^6^,^7^, diverse roles of DBC1 in cellular mechanisms have been introduced and recently DBC1 was re-designated CCAR2 (*c*ell *c*ycle activator and *a*poptosis *r*egulator *2*)^14^,^15^,^16^,^18^,^32^. Moreover, recent report showed that the role of DBC1 in tumorigenesis is SIRT1-independent and primarily involves directly controlling the stabilization of P53^33^. DBC1 stabilized both wild-type P53 and mutant P53. Therefore, DBC1 suppressed proliferation of the cells with wild-type P53, but enhanced the proliferation of the cells with mutant P53^33^. However, siRNA-induced knock-down of DBC1 inhibited proliferation of both NCI-N87 (mutant P53) and MKN-45 (wild-type P53) gastric carcinoma cells^15^. The present study also demonstrated that the knock-down of DBC1 inhibits proliferation and invasion activity of both U2OS (wild-type P53) and SaOS2 (P53-null) cells. In addition, knock-down of DBC1 in P53-null SaOS2 cells increased the expression of P21 and P27 and induced G0/G1 arrest. These findings suggest that DBC1-related regulation of the cell cycle could be P53-dependent and/or P53-independent. In addition, the knock-down of DBC1 decreased expression of invasiveness-related signaling such as NFκB, TGFβ and RhoA, and inhibited the migration and invasion activity of osteosarcoma cells. Similarly, DBC1 is involved in the invasiveness of gastric carcinoma cells by regulating EMT-related signaling^15^ and DBC1 inhibited anoikis by activating the NFκB signaling pathway in breast cancer cells^34^. Moreover, clinically, DBC1 expression was related with invasion and/or metastasis-related clinicopathologic features. There were higher stage tumors and more metastatic tumors in DBC1-expressing osteosarcomas compared with DBC1-negative cases. These results suggest that DBC1 is involved in the progression of osteosarcoma by regulating the invasion-related metastatic potential of osteosarcoma. In line with these results, the immunohistochemical expression of DBC1 was significantly associated with higher tumor stage, higher histologic grade, distant metastasis, and shorter survival in 104 cases of soft-tissue sarcomas^11^. Similarly, the inhibition of invasiveness of squamous carcinoma cells with a knock-down of DBC1 has been reported^17^. However, there are conflicting reports for the role of DBC1 in tumorigenesis. The knock-down of DBC1 with siRNA inhibited cellular stress-induced apoptosis^13^ and enhanced the proliferation of U2OS osteosarcoma cells^35^. Therefore, further study is needed to clarify the exact role of DBC1 in osteosarcoma.

One of the interesting findings from this study and a possible explanation of the tumorigenic role of DBC1 is that DBC1 is involved in the regulation of hormone receptors, especially for the regulation of AR^19^. AR is relatively well-known for its oncogenic role, especially in prostatic carcinomas. However, AR is distributed in a wide-range of tissue types, regardless of sex. The poor prognosis for of patients positive for AR expression has been reported in prostatic cancer^36^, gastric cancer^27^, and hepatocellular carcinoma^37^. In addition, a positive correlation between the expression of AR and DBC1 was seen in clear cell renal cell carcinoma^10^ and diffuse large B cell lymphoma^9^. This study also showed a positive correlation between the protein levels of AR and DBC1 by immunohistochemistry in human osteosarcoma tissues and by western blot in osteosarcoma cells. In two osteosarcoma cell lines, the protein level of AR was affected by the DBC1 expression level, but the expression of DBC1 was not affected by AR expression level. Moreover, in contrast to an earlier report that DBC1 transcriptionally controlled the expression of AR^19^, mRNA level of AR was not affected by a knock-down of DBC1. Only the protein levels of AR decreased with a knock-down of DBC1 and DBC1 has involved in the post-translational stabilization of AR by disturbing ubiquitination and proteosome-mediated degradation of AR. Therefore, our findings suggest that DBC1 is involved in the progression of osteosarcoma by stabilizing AR. In addition, siRNA-mediated knock-down of AR and DBC1 inhibited proliferation and invasion activity of osteosarcoma cells, which correlated with inhibition of proliferation and invasiveness-related signaling. Moreover, overexpression of DBC1 increased proliferation of U2OS cells (Supplementary Fig. S1) and knock-down of DBC1 potentiated the cytotoxicity of doxorubicin (Supplementary Fig. S2). Therefore, present results suggest that suppression of DBC1 and/or AR could be a therapeutic stratagem for the treatment of osteosarcoma patients. In addition, as we have shown in Supplementary Fig. S1, when considering that overexpression of DBC1 could cause the proliferation of osteosarcoma cells despite knock-down of AR, which suggests that DBC1 has its own role independent of the stabilization of AR. Therefore, suppression of DBC1 might be useful for the controlling of osteosarcoma.

In conclusion, this study demonstrated that the expression of DBC1 and AR could be usable as prognostic indicators of osteosarcoma. However, the limitation of this study is that we evaluated only 35 cases of osteosarcomas due to its rarity; however despite the relatively small sample size, we observed statistically significant differences in these cases of osteosarcoma. Therefore, additional study with a large number of cases is needed to confirm the clinical significance of the expression of DBC1 and AR in osteosarcoma patients. However, this study found *in vitro* that DBC1 is important in the post-translational stabilization of AR. Moreover, the inhibition of the proliferation and invasion activity of osteosarcoma cells with the knock-down of DBC1 or AR suggested that the DBC1-AR pathway could be usable as a new therapeutic target in the treatment of osteosarcoma patients. However, due to conflicting reports for the oncogenic role of DBC1 further study is needed.

## Methods

### Osteosarcoma patients and tissue samples

40 cases of primary osteosarcoma patients who underwent surgical resection for the primary lesion between January 1998 and December 2012 were evaluated in this study. Among them, two cases of extra-skeletal osteosarcoma were excluded in this study, and the original histologic slides or tissue blocks were not available in three cases of osteosarcoma of the bone. Finally, we evaluated 35 cases of primary osteosarcoma of the bone, which were reviewed according to the 2013 World Health Organization classification of tumors of soft tissue and bone^1^. The sites of osteosarcomas were the femur (15), the tibia (9), the humorous (7), the maxilla (2), and one each in the rib and the thoracic spine. The median age of the patients was 17 years (range, 6–77 years). Twenty-seven patients received adjuvant chemotherapy, seven patients received postoperative radiation therapy, six patients received both adjuvant chemotherapy and radiation therapy, and 7 patients received no adjuvant treatment. The median follow-up duration was 44 months (range, 4–170 months). The five- and ten-year survival rates were 56% and 51%, respectively. Osteosarcomas were staged based upon the guidelines of the American Joint Committee on Cancer^38^. Clinical information was obtained by reviewing medical records. This study obtained institutional review board approval from Chonbuk National University Hospital (IRB number, CUH 2013-07-036) and written informed consents were obtained from the patients or their legal guardian. All experiments were performed in accordance with relevant guidelines and regulations.

### Immunohistochemical staining and scoring in tissue microarray

To establish a tissue microarray (TMA), we reviewed original H&E slides and took two 3.0 mm cores from each case at the most intact solid areas, mainly composed of tumor cells with the highest histologic grade and no necrosis or degenerative change. The 4 μm thick sections of TMA blocks were incubated with primary antibodies for DBC1 (1:50, Bethyl Laboratories, Mongomery, TX) and AR (1:50, Santa Cruz Biotechnology, Santa Cruz, CA) after microwave antigen retrieval. Immunohistochemical scoring was performed with investigators blinded to the clinicopathological information. Two pathologists (KYJ and KMK) evaluated immunostaining slides for the nuclear staining of DBC1 or AR with consensus under a multi-viewing microscope (Nikon ECLIPSE 80i, Nikon, Tokyo, Japan). The nuclear staining for DBC1 and AR were evaluated by the sum of the staining intensity score (0, no nuclear staining; 1, weak nuclear staining; 2, intermediate nuclear staining; 3, strong nuclear staining) and staining area score (0, no staining cells; 1, 1% of staining cells; 2, 2–10% of staining cells; 3, 11–33% of staining cells; 4, 34–66% of staining cells; 5, 67–100% of staining cells)^39^. Thereafter, the sum scores from the each TMA core were added and used for the final analysis (IHC score)^10^,^11^,^40^. The IHC score ranged from zero to 16.

### Human osteosarcoma cell lines and culture

The human osteosarcoma cell lines, U2OS (wild-type P53) and SaOS2 (P53-null) were purchased from the Korean Cell Line Bank (KCLB, Seoul, Korea) and cultured in DMEM medium supplemented with 10% fetal bovine serum (Gibco BRL, Gaithersburg, MD), penicillin and streptomycin (100 U/ml), and fungizone (0.25 μg/ml, Gibco BRL, Gaithersburg, MD) at 37 °C in humidified incubator with 5% CO_2_.

### Transfection

The short interfering RNA (siRNA) for DBC1 and AR and negative control siRNA duplexes were purchased from Bioneer Corporation (Daejeon, Korea). The first DBC1 duplex (siDBC1 #1) had the sense and antisense sequences 5′-GUGAUGACUAUGACUCCAA-3′ and 5′-UUGGAGUCAUAGUUCAUCAC-3′, respectively. The second DBC1 duplex (siDBC1 #2) had the sense and antisense sequences 5′-GUGUUCUUUGAUGCCAACU-3′ and 5′-AGUUGGCAUCAAAGAACAC-3′, respectively, and the negative control duplex specific had the sense and antisense sequences 5′-CUCAUGGACGGUUGGAUCA-3′ and 5′-UGAUCCAACCGUCCAUGAG-3′, respectively. The first AR duplex (siAR #1) had the sense and antisense sequences 5′-CACAAGUCCCGGAUGUACA-3′ and 5′-UGUACAUCCGGGACUUGUG -3′, respectively. The second AR duplex (siAR #2) had the sense and antisense sequences 5′-CUGAUCUGGUUUUCAAUGA-3′ and 5′-UCAUUGAAAACCAGAUCAG-3′, and the negative control duplex specific had the sense and antisense sequences 5′-CCUACGCCACCAAUUUCGU-3′ and 5′-ACGAAAUUGGUGGCGUAGG-3′, respectively. The plasmid for wild-type DBC1 was kindly provided by L Zannini (Fondazione IRCCS Istituto Nazionale dei Tumori, Italy)^41^. U2OS (3 × 10^5^) and SaOS2 (4 × 10^5^) cells were seeded in 60 mm culture dish in DMEM medium without antibiotic to until they reached 60% confluence and then treated with an siRNA duplex for DBC1 and AR or a plasmid for wild-type DBC1 with Lipofectamine® RNAimax (Invitrogen, Life technologies, Carlsbad, CA) optimized for a 60 mm culture dish following the manufacturer’s protocol.

### Cell proliferation assay

The proliferation of osteosarcoma cell lines was evaluated by an MTT [3-(4,5-dimethylthiazol-2-yl)-2,5-diphenyltetrazolium bromide] assay and a colony forming assay. For the MTT assay, 1 × 10^3^ U2OS cells and 2 × 10^3^ SaOS2 cells were seeded in 96-well plates and the absorbance at 560 nm was measured. Colony forming assays were performed by seeding U2OS (1 × 10^3^ cells/well) and SaOS2 (2 × 10^3^ cells/well) cells in 24 well plates for seven days.

### Trans-chamber migration and invasion assay

The migration assay was performed with 24-transwell migration chambers with 8 μm-pore sized filters (BD Biosciences, San Jose, CA). U2OS (4 × 10^4^) and SaOS2 (1 × 10^5^) cells were seeded in the migration chamber in which the bottom chamber consisted of 1% FBS. For the invasion assay, the same numbers of cells were seeded in the upper chamber containing an 8 μm-pore sized Matrigel Invasion Chamber (BD Biosciences, San Jose, CA). DMEM with 10% FBS was added in the lower chamber in the invasion assay. The migration or invasion chambers were incubated at 37 °C for 24 hours. Thereafter, the lower surface of the filter was stained with DIFF-Quik staining solutions (Sysmex, Japan), and the number of cells which had migrated or invaded the filter were counted in five microscopic fields (magnification x100) per well.

### Cell cycle analysis

The U2OS and SaOS2 cells were fixed in 70% ethanol and treated with DNase-free RNase. The cells were stained with 50 μg/ml propidium iodide (Sigma, St. Louis, MO). Thereafter, cell cycle analysis was performed with a FACStar flow cytometer (Becton-Dickinson, San Jose, CA) and analyzed by CellQuest Pro software (Becton-Dickinson, San Jose, CA).

### Western blotting and immunoprecipitation

The cells were lysed with PRO-PREP Protein Extraction Solution (iNtRON Biotechnology Inc., Korea) and the following antibodies were used for western blot analysis: DBC1 (Bethyl Laboratories, Mongomery, TX), AR (Santa Cruz Biotechnology, Santa Cruz, CA), P53 (Novocastra, Newcastle, UK), acetylated-P53 (Cell Signaling Technology, Beverly, MA), P21 (Santa Cruz Biotechnology, Santa Cruz, CA), P27 (Santa Cruz Biotechnology, Santa Cruz, CA), BAX (Santa Cruz Biotechnology, Santa Cruz, CA), BCL-2 (Santa Cruz Biotechnology, Santa Cruz, CA), TGFβ (Cell Signaling Technology, Beverly, MA), NFκB (Santa Cruz Biotechnology, Santa Cruz, CA), PCNA (Santa Cruz Biotechnology, Santa Cruz, CA), Rho A (Santa Cruz Biotechnology, Santa Cruz, CA), Ubiquitin (Santa Cruz Biotechnology, Santa Cruz, CA), PARP1 (Santa Cruz Biotechnology, Santa Cruz, CA), and actin (Sigma, St. Louis, MO). Western blot images quantified with ImageJ software. For IP, polyclonal anti-DBC1 or anti-AR antibodies were cross-linked to Dynabeads-protein A (Invitrogen, Carlsbad, CA). DBC1 or AR antibodies were immobilized with Dynabeads-protein A for 20 min and then incubated with cell lysates for 1 h at 4 °C. DBC1- or AR-IP complexes were washed three times and the proteins were eluted. Thereafter, we performed western blotting.

### Quantitative reverse-transcription polymerase chain reaction

Total RNA was extracted from cells using an RNeasy Mini Kit (Qiagen Sciences, Valencia, CA). The cDNA was synthesized by using Random 9-mer primers provided with the cDNA synthesis kit (Takara, Kyoto, Japan). Specific primers for DBC1, AR, and GAPDH were designed and checked against the Genbank database (NCBI). The primer sequences were as follows: DBC1/CCAR2; 5′-AAGGGAGACGCCAGAGCAT-3′ and 5′-CATCCAGGGAAGGAGACCAT-3′, AR; 5′-CTGGACACGACAACAACCAG-3′ and 5′-CAGATCAGGGGCGAAGTAGA-3′, and GAPDH; 5′-AACAGCGACACCCACTCCTC-3′ and 5′-GGAGGGGAGATTCAGTGTGG-3′. Quantitative reverse-transcrition polymerase chain reaction was performed in 96 well plates using the Applied Biosystems Prism 7500 Real Time PCR System and SYBR^®^ Green Realtime PCR Master Mix (TOYOBO, Osaka, Japan). All the experiments were performed in triplicate, and the results were normalized to GAPDH expression.

### Immunofluorescence staining

To evaluate the localization of DBC1 and AR in osteosarcoma cells, immunofluorescence staining for DBC1 and AR was performed in U2OS and SaOS2 cells. The cells were fixed with methanol and incubated with antibodies against DBC1 (1:50, clone H-2, Santa Cruz Biotechnology, Santa Cruz, CA) and AR (1:50, clone N-20, Santa Cruz Biotechnology, Santa Cruz, CA) antibody and then incubated with Alexa Fluor^®^ 488 anti-mouse IgG or Alexa Fluor^®^ 594 anti-rabbit IgG (Invitrogen, Carlsbad, CA). The slides were counterstained with DAPI and observed under a Zeiss LSM 510 confocal microscope (Carl Zeiss, Göttingen, Germany).

### Inhibition of ubiquitin proteosomal degradation

The U2OS cells were transfected with DBC1 siRNA as described above. 48 hours after transfection, the cells were treated with 20 μg/ml cycloheximide (CHX; Sigma, St. Louis, MO) or 20 μM MG132 (Sigma, St. Louis, MO) for 0.5 to 8 hours. The cell lysates were blotted with anti-AR antibody (Santa Cruz Biotechnology, Santa Cruz, CA) and anti-actin antibodies. In addition, U2OS cells transfected with control-siRNA or DBC1 siRNA for 24 hours were treated with MG-132 (20 μM) for 4 hours and total lysates of cells were immnunoprecipitated with anti-Androgen receptor antibodies and blotted with anti-Ubiquitin antibodies.

### Statistical analysis

The immunohistochemical staining for DBC1 and AR were grouped as negative or positive with a receiver operating characteristic curve analysis. The end points of interest were OS and RFS. The endpoint of follow-up was the date of last contact or the death of a patient through February 2013. OS was calculated as the period from diagnosis till death from osteosarcoma or last contact. Patients alive at the last contact were censored for OS. RFS was calculated as the time from diagnosis to the date of recurrence, death from osteosarcoma, or last contact of the patient. Those patients who were alive at last contact and had not recurred were censored for RFS. Survival analysis was performed by univariate and multivariate Cox proportional hazards regression analysis and Kaplan-Meier survival analysis with log-rank test. The comparison or relationships between the factors interested in this study were performed by Pearson’s chi-square test or student’s *t*-test. IBM SPSS software (version 20.0) was used in statistical analysis. The *p* values less than 0.05 were considered statistically significant.

## Additional Information

**How to cite this article**: Wagle, S. *et al.* DBC1/CCAR2 is involved in the stabilization of androgen receptor and the progression of osteosarcoma. *Sci. Rep.* **5**, 13144; doi: 10.1038/srep13144 (2015).

## Supplementary Material

Supplementary Information

## Figures and Tables

**Table 1:** Clinicopathologic variables and the expression status of DBC1 and androgen receptor in 35 osteosarcoma patients.

Characteristics		N	DBC1		AR	
			positive	*p*	positive	*p*
Age, yr	<30	25	11 (44%)	0.392	12 (48%)	0.915
	≥30	10	6 (60%)		5 (50%)	
Sex	Female	10	1 (10%)	0.004	3 (30%)	0.164
	Male	25	16 (64%)		14 (56%)	
Tumor size, cm	≤8	17	5 (29%)	0.028	8 (47%)	0.862
	>8	18	12 (67%)		9 (50%)	
Stage	I	11	2 (18%)	0.035	1 (9%)	0.005
	II	19	11 (58%)		12 (63%)	
	III–IV	5	4 (80%)		4 (80%)	
Distant metastasis	Absence	30	13 (43%)	0.129	13 (43%)	0.129
	Presence	5	4 (80%)		4 (80%)	
Histological grade	Low	11	2 (18%)	0.015	1 (9%)	0.002
	High	24	15 (63%)		16 (67%)	
Histologic type	Conventional	31	16 (52%)	0.316	14 (45%)	0.261
	Non-conventional	4	1 (25%)		3 (75%)	
AR	Negative	18	4 (22%)	0.001		
	Positive	17	13 (76%)			

DBC1, deleted in breast cancer 1; AR, androgen receptor.

**Table 2:** Clinicopathologic factors and their effect on overall survival and relapse-free survival by univariate and multivariate Cox proportional hazards regression analysis.

Characteristics	N	OS		RFS	
		HR (95% CI)	*p*	HR (95% CI)	*p*
Univariate analysis					
Age, yr, ≥30 (*vs.* <30)	10/35	2.507 (0.886–7.093)	0.083	2.810 (1.039–7.598)	0.042
Sex, male (*vs.* female)	25/35	1.684 (0.470–6.028)	0.423	2.025 (0.567–7.237)	0.278
Tumor size, cm, >8 (*vs.* ≤8)	18/35	3.591 (1.126–11.459)	0.031	3.318 (1.131–9.737)	0.029
Stage, I	11/35	1	0.024	1	0.027
II	19/35	8.090 (1.041–62.838)	0.046	8.933 (1.157–68.974)	0.036
III–IV	5/35	23.521 (2.382–232.242)	0.007	23.559 (2.338–237.356)	0.007
Distant metastasis, presence (*vs.* absence)	5/35	4.551 (1.237–16.748)	0.023	4.078 (1.097–15.162)	0.036
Histological grade, high (*vs.* low)	24/35	9.335 (1.223–71.232)	0.031	10.022 (1.317–76.243)	0.026
DBC1, positive (*vs.* negative)	17/35	8.639 (1.940–38.469)	0.005	6.657 (1.875–23.629)	0.003
AR, positive (*vs.* negative)	17/35	5.551 (1.551–19.864)	0.008	4.201 (1.336–13.216)	0.014
Multivariate analysis Model 1*					
Age, yr, ≥30 (*vs.* <30)				2.652 (0.971–7.239)	0.057
DBC1, positive (*vs.* negative)		8.639 (1.940–38.469)	0.005	6.555 (1.825–23.549)	0.004
Multivariate analysis Model 2**					
Age, yr, ≥30 (*vs.* <30)				2.864 (1.017–8.066)	0.046
Tumor size, cm, >8 (*vs.* ≤8)		3.891 (1.169–12.950)	0.027	3.777 (1.205–11.846)	0.023
AR, positive (*vs.* negative)		5.892 (1.616–21.480)	0.007	3.975 (1.194–13.238)	0.025

OS, overall survival; RFS, relapse-free survival; HR, hazard ratio; 95% CI, 95% confidence interval; DBC1, deleted in breast cancer 1; AR, androgen receptor.

^*^Variables considered in the multivariate analysis Model 1 were the age, tumor size, tumor stage, distant metastasis, histologic grade, DBC1 expression, and AR expression.

^**^Variables considered in the multivariate analysis Model 2 were the age, tumor size, tumor stage, distant metastasis, histologic grade, and AR expression.
