# Supplementary material for: DBC1/CCAR2 is involved in the stabilization of androgen receptor and the progression of osteosarcoma
Source: Sci Rep. 2015 Aug 7;5:13144. doi: 10.1038/srep13144 (PMC4642542; doi:10.1038/srep13144)
Supplement: Supplementary Information [file srep13144-s1.pdf]

Supplementary information

DBC1/CCAR2 is involved in the stabilization of androgen receptor and the progression of osteosarcoma

Sajeev Wagle[1,#], See-Hyoung Park[2, #], Kyoung Min Kim[3], Young Jae Moon[1], Jun Sang Bae[3], Keun Sang Kwon[4], Ho Sung Park[3], Ho Lee[5], Woo Sung Moon[3], Jung Ryul Kim[1,*] & Kyu Yun Jang[3,*]

[1]Department of Orthopedic Surgery, Chonbuk National University Medical School, Research Institute of Clinical Medicine of Chonbuk National University-Biomedical Research Institute of Chonbuk National University Hospital and Research Institute for Endocrine Sciences, Jeonju, Republic of Korea;

[2]Program in Nano Science and Technology, Department of Transdisciplinary Studies, Seoul National University Graduate School of Convergence Science and Technology, Suwon, Korea;

[3]Department of Pathology, Chonbuk National University Medical School, Research Institute of Clinical Medicine of Chonbuk National University-Biomedical Research Institute of Chonbuk National University Hospital and Research Institute for Endocrine Sciences, Jeonju, Republic of Korea;

[4]Department of Preventive Medicine, Chonbuk National University Medical School, Jeonju, Republic of Korea;

[5]Department of Forensic Medicine, Chonbuk National University Medical School, Jeonju, Republic of Korea

[#]These authors contributed equally to this work.

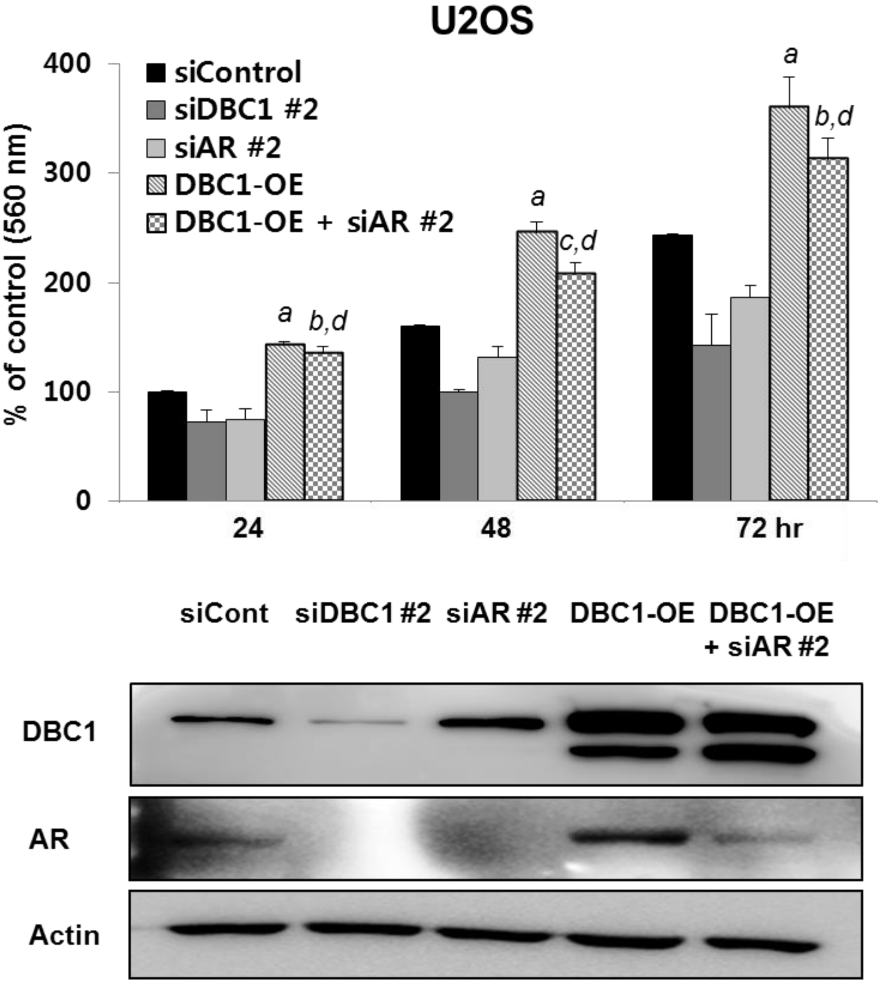

Supplementary Figure S1. The effect of the co-transfection of wild-type DBC1 plasmid and siRNA for AR for the proliferation of U2OS cell. The knock-down of DBC1 or AR with siRNA for DBC1 or AR inhibit the proliferation of U2OS cells as indicated by an MTT assay. The proliferation of U2OS cell was significantly increased with induced overexpression of DBC1 compared with control cells (*a*; *versus* control cells, $p < 0.001$). The proliferation of U2OS cell was significantly decreased with co-transfection of siRNA for AR in the U2OS cells induced to overexpress DBC1 compared with the cell induced to overexpress DBC1 (*b*; *versus* DBC1 overexpression, $p < 0.05$, *c*; *versus* DBC1 overexpression, $p < 0.001$). However, the proliferation of the co-transfected cells was significantly higher than the control cells (*d*; *versus* control cells, $p < 0.001$).

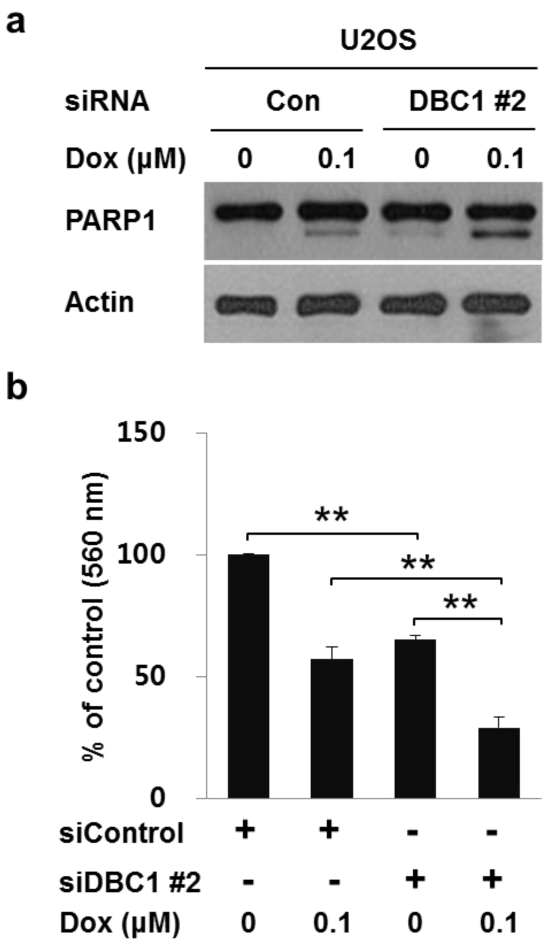

Supplementary Figure S2. Attenuation of DBC1 induces PARP1 cleavage and sensitizes U2OS cells to doxorubicin. U2OS cells were transfected with control or DBC1 siRNA and treated with doxorubicin (0.1 μM, Sigma, St. Louis, MO) for 24 hrs. (a) The cells were lysed and subjected to immunoblotting with PARP1 antibodies and β-actin was used for a loading control. PARP1 cleavage significantly increased with the treatment of doxorubicin or knock-down of DBC1. Moreover, knock-down of DBC1 potentiated PARP1 cleavage induced by doxorubicin. (b) The treatment of doxorubicin or knock-down of DBC1 showed significant cytotoxicity. In addition, knock-down of DBC1 potentiated the cytotoxicity induced by doxorubicin. Statistical analysis performed by one-way ANOVA with Tukey HSD test. **, $p < 0.001$.
